# Development of an efficient gene-targeting system for elucidating infection mechanisms of the fungal pathogen *Trichosporon asahii*

**DOI:** 10.1038/s41598-021-97287-3

**Published:** 2021-09-14

**Authors:** Yasuhiko Matsumoto, Tae Nagamachi, Asami Yoshikawa, Hideki Yamazaki, Yusuke Yamasaki, Tsuyoshi Yamada, Takashi Sugita

**Affiliations:** 1 Department of Microbiology, Meiji Pharmaceutical University, 2-522-1, Noshio, Kiyose, Tokyo 204-8588 Japan; 2 Teikyo University Institute of Medical Mycology, 359 Otsuka, Hachioji, Tokyo 192-0395 Japan; 3 Asia International Institute of Infectious Disease Control, Teikyo University, 2-11-1, Kaga, Itabashi-ku, Tokyo, 173-8605 Japan

**Keywords:** Gene targeting, Microbiology techniques, Fungal genetics, Fungal pathogenesis

## Abstract

*Trichosporon asahii* is a pathogenic fungus that causes severe, deep-seated fungal infections in neutropenic patients*.* Elucidating the infection mechanisms of *T. asahii* based on genetic studies requires a specific gene-targeting system. Here, we established an efficient gene-targeting system in a highly pathogenic *T. asahii* strain identified using the silkworm infection model. By comparing the pathogenicity of *T. asahii* clinical isolates in a silkworm infection model, *T. asahii* MPU129 was identified as a highly pathogenic strain. Using an *Agrobacterium tumefaciens*-mediated gene transfer system, we obtained a *T. asahii* MPU129 mutant lacking the *ku70* gene, which encodes the Ku70 protein involved in the non-homologous end-joining repair of DNA double-strand breaks. The *ku70* gene-deficient mutant showed higher gene-targeting efficiency than the wild-type strain for constructing a mutant lacking the *cnb1* gene, which encodes the beta-subunit of calcineurin. The *cnb1* gene-deficient mutant showed reduced pathogenicity against silkworms compared with the parental strain. These results suggest that an efficient gene-targeting system in a highly pathogenic *T. asahii* strain is a useful tool for elucidating the molecular mechanisms of *T. asahii* infection.

## Introduction

*Trichosporon asahii* is a basidiomycete yeast that is widely distributed in the environment and is often isolated from human blood, sputum, skin, feces, and urine^1–6^. *T. asahii* causes severe, deep-seated fungal infections in neutropenic patients^7–9^. Deep mycoses caused by *T. asahii* has a twofold higher mortality rate than those caused by *Candida albicans* (80% vs 40%)^10^. Since *T. asahii* is resistant to echinocandin antifungals, patients treated with micafungin are susceptible to the development of severe infections^11^. *T. asahii* strains resistant to amphotericin B and azole antifungals such as fluconazole have also been isolated from patients^12,13^. Moreover, *T. asahii* forms a biofilm, a three-dimensional structure comprising microbe aggregates and extracellular matrix, on catheter surfaces in patients^14^. The *T. asahii* cells within biofilms are resistant to antifungal drugs^13^. *T. asahii* has morphological forms: yeast form, hyphae (filament form) and arthroconidia (chains of cells and asexual spores)^4^. Furthermore, arthroconidia of *T. asahii* may play a key role in biofilm formation by promoting cellular adhesion^15^. *T. asahii* is therefore a highly problematic clinical pathogen^9^. Since the technology to construct gene-deficient mutants of *T. asahii* has not been established, it has not been possible to study pathogenicity and drug resistance in *T. asahii* using a gene-deficient mutant.

In general, mammals such as mice are used as experimental models in studies of infectious diseases^16^. The use of mammalian animals in infection experiments requires specialized experimental facilities, and the large number of animals required for these studies is a severe limitation due to ethical issues regarding animal welfare^17^. *T. asahii* infection experiments are not easy to perform in mice because immunosuppressive drugs must be administered^18,19^. To address these issues, we established a silkworm infection model for elucidating the mechanisms of *T. asahii* infection^20^. Compared with mammals such as mice, the use of invertebrate silkworms is advantageous because they are less costly to house and easier to rear in large numbers in simple facilities, and fewer ethical problems are associated with their use. Therefore, the use of silkworms as an experimental animal enhances the feasibility of performing large-scale, in vivo screening using a large number of individuals^16^.

Novel virulence genes in the pathogenic bacterium *Staphylococcus aureus* were identified using a silkworm infection model and a library of gene-deficient strains^21,22^. Silkworm infection models have also been used to identify virulence genes of the pathogenic fungi *C. albicans* and *Candida glabrata*^23,24^. In *Cryptococcus neoformans*, a basidiomycete yeast like *T. asahii*, a strain that is highly pathogenic to mice is also highly pathogenic to silkworms^25^. Moreover, a *C. neoformans* strain lacking the gene encoding the calcineurin subunit, which contributes to the pathogenicity against mice, was less virulent against silkworms^25^. Since the silkworm can be used to evaluate differences in the pathogenicity between strains of pathogenic fungi, the silkworm infection model with *T. asahii* may be useful for elucidating the infection mechanisms of *T. asahii* with the gene-deficient strains. We successfully established a *T. asahii* strain that expresses green fluorescent protein using an *Agrobacterium tumefaciens*-mediated gene transfer (ATMT) system^20^. A method for generating a gene-deficient strain of *T. asahii*, however, has not yet been established.

Homologous recombination (HR), a repair mechanism for DNA double-strand breaks (DSBs), is required to introduce mutations into a gene-targeting system using homologous DNA fragments^26,27^. Another repair mechanism is the non-homologous end joining (NHEJ) of DSBs^26^. These 2 main repair mechanisms affect gene-targeting efficiency by introducing homologous DNA fragments. NHEJ repair mediates the insertion of introduced homologous DNA fragments into genome sites that are different from the target region, thereby reducing the gene-targeting efficiency for generating a gene-deficient strain^26^. Therefore, gene-targeting efficiency can be increased by inhibiting NHEJ repair^26,28^. Ku70 and Ku80 proteins form heterodimers and are involved in the NHEJ repair for DSBs^29^. In several fungi, deletion of the genes encoding these proteins led to the increase of gene-targeting efficiency for generating gene-deficient strains^28,30,31^. In *C. neoformans*, gene-deficient strains could be generated in the *ku80* gene-deficient strain, but not in the wild-type strain, by electroporation, a gene transfer method^32^. Therefore, strains with inhibited NHEJ repair due to disruption of the gene encoding Ku proteins are useful parental strains for promoting genetic studies.

In this study, we identified the *T. asahii* MPU129 strain, a clinical isolate that is highly pathogenic to silkworms, and generated a *T. asahii* MPU129 mutant deficient in the *ku70* gene. Gene-targeting efficiency to obtain the *ku70* gene-deficient strain was higher than that in the wild-type strain. Our findings suggest that a *T. asahii* strain showing high gene-targeting efficiency and the silkworm infection model are useful tools for studying infectious diseases as a preliminary step to conducting experiments in mice.

## Results

### Comparison of the pathogenicity of *T. asahii* strains using a silkworm infection model

**Figure 1:**
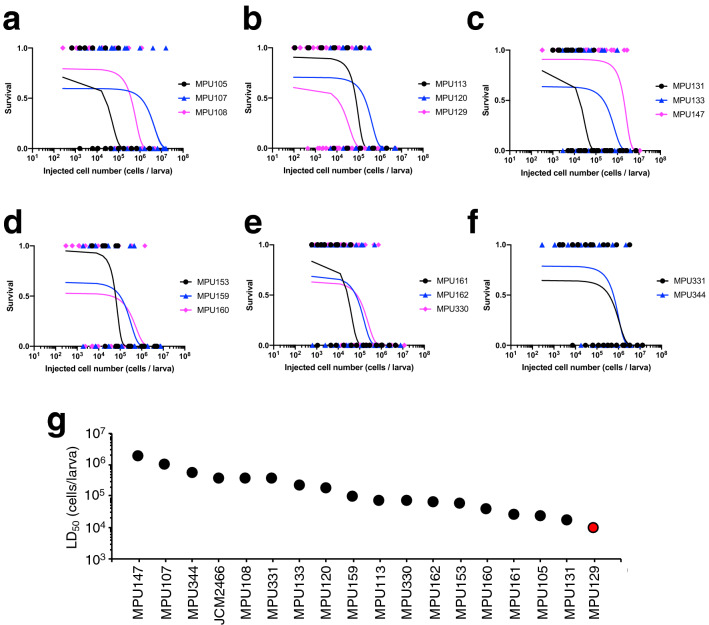
Comparison of pathogenicity of *T. asahii* strains against silkworms. (**a–f**) The number of surviving silkworms under a rearing condition at 37 °C was determined 48 h after administration of the fungal cells (1 × 10^2^ to 2 × 10^7^ cells/larva) into the hemolymph of silkworms. Survived and dead silkworms are indicated as 1 and 0, respectively. n = 4/group. The curves were drawn from the combined data of 2–6 independent experiments by a simple logistic regression model. (**g**) LD_50_ values of *T. asahii* strains. Data for JCMM2466 are cited from Matsumoto et al.^20^.

Highly pathogenic strains are useful for understanding the molecular mechanisms of pathogens because several pathogenic strains obtain virulence genes by horizontal gene transfer and gene mutation^33,34^. First, we identified *T. asahii* strains that are highly pathogenic to silkworms. Using a silkworm infection model, we determined the median lethal dose (LD_50_ values) on the basis of curves drawn by a simple logistic regression model (Fig. 1). The LD_50_ values of the 17 clinical isolates were 9.3 × 10^3^–2.0 × 10^6^ cells/larva and the LD_50_ value of the MPU129 strain was the lowest, more than tenfold lower than that of the JCM2466 strain (Fig. 1g). The result suggests that the pathogenicity of the MPU129 strain against silkworms is highest among these *T. asahii* strains.

### Generation of the *ku70* gene-deficient mutant in the *T. asahii* MPU129 strain

**Figure 2:**
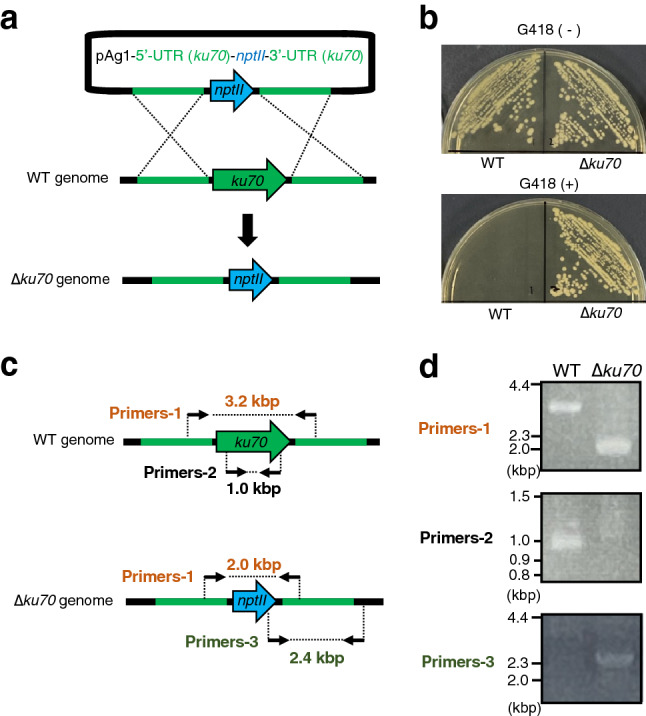
Construction of the *ku70* gene-deficient mutant in the *T. asahii* MPU129 strain by the ATMT system. (**a**) Structure of the plasmid used to construct the *ku70* gene-deficient mutant and the predicted genome of the *ku70* gene-deficient mutant. (**b**) The wild-type (MPU129) and *ku70* gene-deficient candidate were spread on Sabouraud agar medium with or without G418 (50 µg/ml) and incubated at 27 °C for 2 days. (**c**) Location of the primers for confirming the genome structure of the *ku70* gene-deficient candidate by PCR. (**d**) Confirmation of the *ku70* gene-deficiency of the *ku70* gene-deficient candidate by PCR using extracted genome DNA. Cropped blots were used. Full-length blots are presented in Supplementary Fig. 1.

We next obtained a *ku70* gene-deficient mutant of the MPU129 strain using the ATMT system. The targeting plasmid, pAg1-5′UTR (*ku70*)-*nptII*-3′UTR (*ku70*), contained the *nptII* gene that leads to resistance against G418, an aminoglycoside used as a selective agent for eukaryotic cells (Fig. 2a). Colonies on Sabouraud dextrose agar containing G418 were obtained using the ATMT system (Fig. 2b). In the genome of the 414th candidate colony, polymerase chain reaction (PCR) amplification revealed DNA fragments of the predicted size (Fig. 2c,d). The results suggest that the *ku70* gene-deficient mutant in the *T. asahii* MPU129 strain was obtained using the ATMT system.

### Effect of *ku70* gene deficiency on growth and pathogenicity of the *T. asahii* MPU129 strain

**Figure 3:**
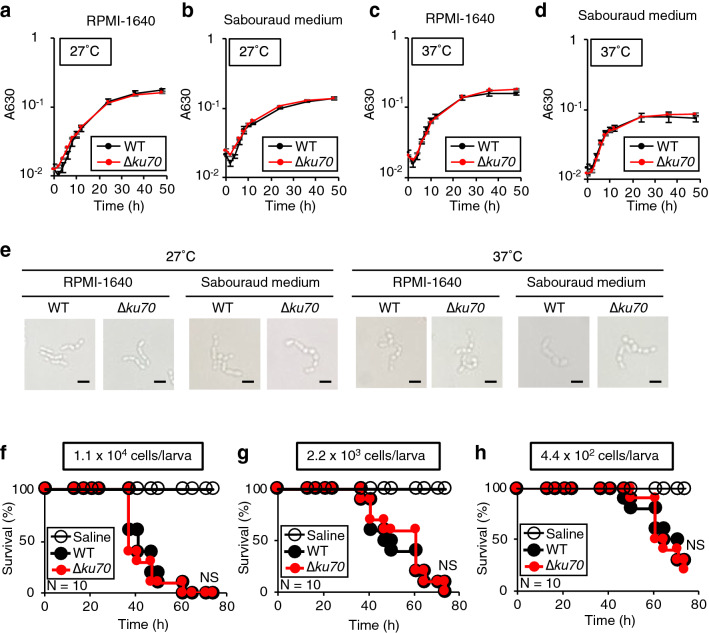
Effects of *ku70* gene-deficiency in the *T. asahii* MPU129 mutant on growth, morphology, and pathogenicity against silkworms. (**a–d**) The wild-type (MPU129) and *ku70* gene-deficient mutant were inoculated on RPMI-1640 medium and Sabouraud medium and incubated at 27 °C or 37 °C. Absorbance 630 nm of the culture was monitored. Data are shown as means ± standard error of the mean (SEM). (**e**) The wild-type (MPU129) and *ku70* gene-deficient mutant were incubated at 2 days after inoculation and observed with a microscope. (**f**–**h**) Saline, *T. asahii* MPU129 (WT), or the *ku70* gene-deficient mutant (∆*ku70*) [1.1 × 10^4^ cells/larva (**f**), 2.2 × 10^3^ cells/larva (**g**), 4.4 × 10^2^ cells/larva (**h**)] were injected into the silkworm hemolymph and incubated at 37 °C. The survival of the silkworms was monitored for 74 h. The significance of differences between groups in silkworm infection experiments was calculated by the log-rank test based on the curves by the Kaplan–Meier method. *NS* Not significant (*P* > 0.05). n = 10/group.

We investigated whether the deficiency of *ku70* gene in the *T. asahii* MPU129 strain affected its growth on nutrient media and its pathogenicity in silkworms. The growth of the *ku70* gene-deficient mutant in RPMI-1640 or Sabouraud liquid medium was similar to that of wild-type at either 27 °C or 37 °C (Fig. 3a–d). Moreover, microscopic analysis did not reveal significant differences in the morphology (Fig. 3e). Furthermore, the time required for the *ku70* gene-deficient mutant to kill all the silkworms was similar to that of the wild-type strain (Fig. 3f–h). These results demonstrated that *ku70* gene deficiency in the *T. asahii* MPU129 strain did not significantly affect its growth on nutrient media or its pathogenicity to silkworms.

### Increased gene-targeting efficiency in the *ku70* gene-deficient *T. asahii* mutant

**Figure 4:**
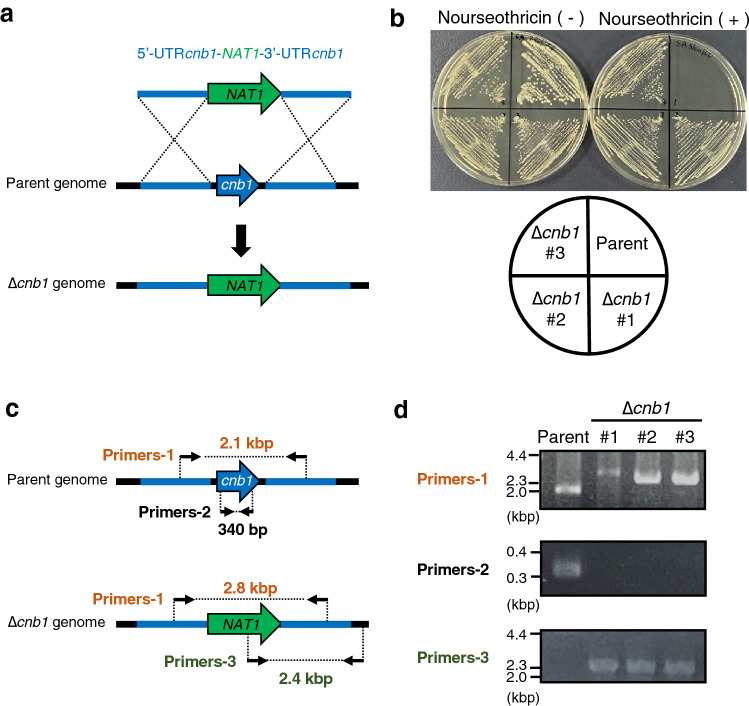
Construction of *cnb1* gene-deficient mutant in the *ku70* gene-deficient mutant of *T. asahii* MPU129 strain by electroporation. (**a**) Structure of the DNA fragment for construction of the *cnb1* gene-deficient mutant and the predicted genome of the *cnb1* gene-deficient mutant. (**b**) The parent strain (MPU129 ∆*ku70*) and *cnb1* gene-deficient candidates (∆*cnb1* #1, #2, and #3) were spread on Sabouraud agar medium with or without nourseothricin (100 µg/ml) and incubated at 27 °C for 2 days. (**c**) Location of the primers for confirming the genome structure of the *cnb1* gene-deficient candidate by PCR. (**d**) Confirmation of the *cnb1* gene-deficiency of the *cnb1* gene-deficient candidate by PCR using extracted genome DNA. Cropped blots were used. Full-length blots are presented in Supplementary Fig. 1.

We examined whether gene-targeting efficiency was increased in the *ku70* gene-deficient mutant by determining the ratio of the strain lacking the *cnb1* gene, which encodes the β-subunit of calcineurin. Since gene-targeting efficiency in *ku80* gene-deficient mutant of *C. neoformans* was tested by electroporation, a faster and simpler gene transfer method^32^, we also used electroporation to investigate *T. asahii*. A DNA fragment, 5'UTR (*cnb1*)-*NAT1*-3'UTR (*cnb1*), was introduced to delete the *cnb1* gene by electroporation. Nourseothricin-resistant strains were obtained, and each colony was confirmed by PCR to be deficient in the *cnb1* gene (Fig. 4a–d). Of the 21 nourseothricin-resistant colonies obtained by introducing the 5'UTR (*cnb1*)-*NAT1*-3'UTR (*cnb1*) into the *ku70* gene-deficient mutant, 4 were deficient for the *cnb1* gene (Table 1). On the other hand, none of the 120 nourseothricin-resistant colonies obtained by introducing the 5'UTR (*cnb1*)-*NAT1*-3′UTR (*cnb1*) into the wild-type was deficient for the *cnb1* gene (Table 1). These results suggest that the deficiency of the *ku70* gene in the MPU129 strain increases the gene-targeting efficiency for generating a gene-deficient mutant by electroporation.

**Table 1:** Efficiency of homologous replacement on *cnb1* gene region.

Strain	Total transformants	Homologous replacement (∆*cnb1*)	Efficiency (%) (∆*cnb1*/total transformants)
Wild type	120	0	0%
∆*ku70*	21	4	19%

### Attenuated pathogenicity of the *cnb1* gene-deficient mutant against silkworms

**Figure 5:**
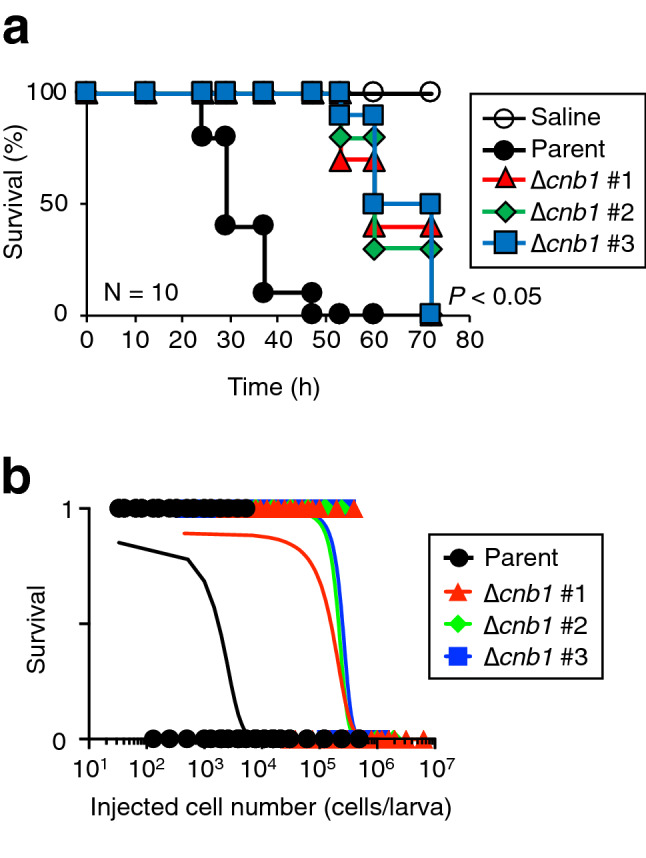
Attenuated pathogenicity of *T. asahii* against silkworms by *cnb1* gene-deficiency. (**a**) Saline, *T. asahii* MPU129 ∆*ku70* (parent strain) (5.4 × 10^4^ cells/larva), or the *cnb1* gene-deficient mutants [∆*cnb1* #1 (7.1 × 10^4^ cells/larva), #2 (5.2 × 10^4^ cells/larva), and #3 (2.5 × 10^4^ cells/larva)] were injected into the silkworm hemolymph and the silkworms were incubated at 37 °C. The survival of the silkworms was monitored for 72 h. The significance of differences between parent strain group and the *cnb1* gene-deficient mutant groups was calculated by the log-rank test based on the curves by the Kaplan–Meier method. n = 10/group. (**b**) The number of surviving silkworms under a rearing condition at 37˚C was determined at 48 h after administration of the fungal cells (3.3 × 10^2^ to 6.2 × 10^6^ cells/larva) into the hemolymph of silkworms. Survived and dead silkworms were indicated as 1 and 0, respectively. n = 4/group. The curves were drawn from combined data of 2–3 independent experiments by simple logistic regression model.

In *C. neoformans*, the pathogenicity of the *cnb1* gene-deficient mutant against silkworms was reduced^25^. We examined whether the *cnb1* gene-deficient mutants of *T. asahii* had reduced pathogenicity against silkworms. The survival time of silkworms injected with the *cnb1* gene-deficient mutants was longer than that of the parental strain (Fig. 5a). The LD_50_ values of the *cnb1* gene-deficient mutants were 89-fold higher than that of the parent strain (Fig. 5b). The result suggests that pathogenicity against silkworms was reduced by *cnb1* gene deficiency in *T. asahii*.

## Discussion

In this study, we identified a *T. asahii* strain that is highly pathogenic against silkworms and established a platform for generating a gene-deficient mutant. The *cnb1* gene-deficient mutant obtained using the technique showed decreased pathogenicity against silkworms. To our knowledge, this is the first report of a method for obtaining a gene-deficient mutant of *T. asahii*. Our results suggest that the calcineurin pathway is involved in the pathogenicity of *T. asahii*.

In the silkworm infection model with *T. asahii*, the MPU129 strain showed high pathogenicity among clinical isolates used in this study. We assumed that the MPU129 strain can adapt to the host environments and appropriately regulate the pathogenicity compared with other isolates. To reveal the relationship between clinical information and the pathogenicity in the silkworm infection model among the clinical isolates will be an important study.

Silkworms are suitable experimental animals for performing large-scale in vivo evaluations because they are relatively inexpensive and few ethical issues are associated with their use. Therefore, silkworm infection models are suitable for quantitative evaluation of the pathogenicity of microorganisms based on the calculation of LD_50_ values. Using the silkworm infection model and a gene-deficient mutant library of *S. aureus*, we previously identified the virulence genes that contribute to pathogenicity against mice^21^. The gene-targeting system in *T. asahii* developed in the present study will facilitate the construction of a gene-deficient mutant library of *T. asahii*. It is expected that a gene-deficient mutant library of *T. asahii* for application to silkworm infection models will help elucidate the molecular mechanisms of *T. asahii* infection. The contribution of candidate virulence genes to pathogenicity that is identified using the silkworm infection model should be confirmed by infection experiments in mice.

Gene-targeting efficiency by electroporation was higher in the *ku70* gene-deficient mutant, while growth on nutrient media and pathogenicity to silkworms remained unaltered. Therefore, the *ku70* gene-deficient mutant is useful as a parental strain for elucidating the infection mechanism of *T. asahii* based on genetic studies. In the pathogenic fungus *Aspergillus fumigatus*, the pathogenicity of the *ku80* gene-deficient mutant did not differ from that of wild-type against mice^35^. We considered that NHEJ repair of DSBs might not be greatly involved in the pathogenicity of *T. asahii* or *A. fumigatus*. Although electroporation is a faster and simpler gene transfer method than the ATMT system for obtaining gene-deficient mutants, homologous recombination by electroporation occurs at low frequency^32,36^. When using electroporation, no *cnb1* gene-deficient mutants were obtained in the wild-type, but a 19% ratio of mutants was obtained in the *ku70* gene-deficient mutant. The result suggests that NHEJ of DSBs occurs at a high frequency in *T. asahii*. Therefore, we reasoned that the generation of target gene-deficient mutants by electroporation requires the *ku70* gene-deficient mutant that lacks NHEJ repair activity. When we obtain fungal colonies grown on a drug-containing agar medium, both strains with mutations in the targeted gene region and strains with non-specific gene insertions caused by NHEJ were obtained. Therefore, NHEJ may contribute to obtaining the strains with a non-specific gene inserted mutants by selecting drug-resistant strains. It can be constructed the gene-deficient mutants in *T. asahii* within two weeks by using the TR129 *ku70* gene-deficient mutant with electroporation method. Construction of a gene-deficient mutant library of *T. asahii* using the *ku70* gene-deficient mutant as a parent strain is thus planned for future studies.

Although we tried to obtain a *cnb1* gene-deficient mutant using the *ku70* gene-deficient mutant as the parent strain with the ATMT system, we did not obtain a drug-resistant candidate. Optimization of the ATMT system using the *ku70* gene-deficient mutant is needed to obtain target gene-deficient mutants. Moreover, CRISPR-CAS9 technology was applied for gene editing in fungi including *C. neoformans*^37,38^. The establishment of the CRISPR-CAS9 mediated gene-editing method for *T. asahii* will be a future subject.

In *C. neoformans*, the calcineurin pathway is involved in capsule production and melanin synthesis, which are responsible for evading host immunity^39^. A calcineurin-deficient strain of *C. neoformans* showed decreased pathogenicity against mice and silkworms^25,40,41^. The present study also showed that the calcineurin pathway is involved in the pathogenicity of *T. asahii* against silkworms. The calcineurin in *C. neoformans* regulates gene expression via the dephosphorylation of the transcription factors^39^. Therefore, we assumed that the calcineurin in *T. asahii* also regulates the virulence-related gene expression. To reveal the role of calcineurin in the *T. asahii* pathogenicity will be an important subject. Further studies are needed to investigate the generation of a revertant strain obtained by reintroducing the *cnb1* gene into the *cnb1* gene-deficient mutant and to perform a detailed functional analysis using the *cnb1* gene-deficient mutant and its revertant strain.

In conclusion, we established a simple method for generating a gene-deficient *T. asahii* strain that is highly pathogenic against silkworms. The *ku70* gene-deficient mutant in the *T. asahii* MPU129 strain is useful as a parental strain for genetic studies and an important tool for studying infectious diseases of *T. asahii*.

## Methods

### Reagents

Kanamycin, cefotaxime, and chloramphenicol were purchased from Wako Pure Chemical Industries (Osaka, Japan). Nourseothricin and G418 were purchased from Jena Bioscience (Dortmund, Germany) and Enzo Life Science, Inc. (Farmingdale, NY, USA), respectively.

### Culture of *T. asahii*

The *T. asahii* strains (MPU105, MPU107, MPU108, MPU113, MPU120, MPU129, MPU131, MPU133, MPU147, MPU153, MPU159, MPU160, MPU161, MPU162, MPU330, MPU331, and MPU344) used in this study were stocked in the previously reported MPU library^15^. *T. asahii* strains were grown on Sabouraud dextrose agar (1% hipolypepton [Nihon Pharmaceutical Co., Ltd., Tokyo, Japan], 4% dextrose and 1.5% agar [both from FUJIFILM Wako Pure Chemical Industries, Osaka, Japan]) and incubated at 27 °C for 2 days.

For growth on liquid medium, RPMI-1640 (RPMI medium 1640 [Life Technologies Ltd., Paisley, UK] containing 165 mM 3-(*N*-morpholino) propanesulfonic acid [Dojindo Laboratories, Kumamoto, Japan], pH 7.0) and Sabouraud liquid medium (1% hipolypeptone, 4% dextrose) were used in this study. Suspensions of wild-type *T. asahii* (MPU129 strain) and *ku70* gene-deficient *T. asahii* mutants were prepared with RPMI-1640 or Sabouraud medium and adjusted to 0.01–0.02 on absorbance at 630 nm. The *T. asahii* suspensions were incubated at 27 °C or 37 °C for 48 h and absorbance at 630 nm was measured using a microplate reader (iMark™ microplate reader; Bio-Rad Laboratories Inc., Hercules, CA, USA). After incubation for 2 days, the *T. asahii* cells were observed with a light microscope (CH30; Olympus, Tokyo, Japan).

### Silkworm infection experiments

Silkworm infection experiments were performed according to a previous report^20^. Eggs of silkworms (Hu・Yo × Tukuba・Ne) were purchased from Ehime-Sanshu Co., Ltd. (Ehime, Japan), disinfected, and hatched at 25–27 °C. The silkworms were fed an artificial diet, Silkmate 2S, containing antibiotics purchased from Ehime-Sanshu Co., Ltd. Fifth instar larvae were used in the infection experiments. Silkworm fifth instar larvae were fed the artificial diet (Silkmate 2S; Ehime-Sanshu Co., Ltd.) overnight. *T. asahii* grown on Sabouraud agar plates was suspended in physiologic saline solution (0.9% w/v NaCl) and filtered through a 40-μm cell strainer (Corning Inc., Corning, NY, USA). A 50-µl suspension of *T. asahii* cells was administered into the silkworm hemolymph by injecting the silkworm dorsally using a 1-ml tuberculin syringe (Terumo Medical Corporation, Tokyo, Japan). Silkworms injected with *T. asahii* cells were placed in an incubator and their survival was monitored.

### LD_50_ measurement

The dose of *T. asahii* required to kill half of the silkworms (LD_50_) was determined according to the previous report^20^. *T. asahii* strains (1 × 10^2^ to 2 × 10^7^ cells/50 µl) were injected into the silkworm hemolymph and the silkworms were incubated at 37 °C. Survival of the silkworms (n = 4/group) at 48 h was monitored. The LD_50_ was determined from the combined data of 2–3 independent experiments by simple logistic regression model using Prism 9.1.2 (GraphPad Software, LLC, San Diego, CA, USA, https://www.graphpad.com/scientific-software/prism/).

### Construction of gene-deficient mutants in *T. asahii*

The plasmid for gene-deficient mutants in *T. asahii* was constructed according to a previous report^30^. To generate the *ku70* gene-deficient mutant, the 5′-UTR of the *ku70* gene, neomycin phosphotransferase gene (*nptII*) cassette, and 3′-UTR of the *ku70* gene were introduced into a pAg1 vector^42^. To generate the *cnb1* gene-deficient mutant, the 5′-UTR and 3′-UTR of the *cnb1* gene were introduced into a pAg1-NAT1 vector^42^. Cloning was performed by the infusion method according to the general method (In-Fusion HD Cloning Kit, Takara, Shiga, Japan). The primers used for PCR amplification of each DNA region are shown in Table 2. The pAg1-5′UTR(*ku70*)-*nptII*-3′UTR(*ku70*) was introduced into the *T. asahii* MPU129 strain using the *A. tumefaciens*-mediated transformation method described previously^20^. The pAg1-5′UTR(*ku70*)-*nptII*-3′UTR(*ku70*) was introduced into the *A. tumefaciens* EHA105 strain by electroporation and transformants were grown on 2 × YT agar (1.6% tryptone and 1% yeast extract [both from Becton, Dickinson, and Company, NJ, USA], 0.5% NaCl, and 1.5% agar) containing chloramphenicol (25 μg/ml) and kanamycin (50 μg/ml). The *A. tumefaciens* EHA105 strain harboring the plasmid introduces the 5′UTR(*ku70*)-*nptII*-3′UTR(*ku70*) fragment into the nucleus of *T. asahii* cells via the DNA transport proteins. The transformant was co-cultured with the *T. asahii* MPU129 strain at 27 °C for 2 days. The candidates were isolated as colonies grown on Sabouraud dextrose agar containing G418 (50 μg/ml) and cefotaxime (100 μg/ml). Introduction of the mutation into the genome of the candidate strains was confirmed by PCR using the primers shown in Table 2.

**Table 2:** Primers used in this study.

Primers	Nucleic acid sequence
**pAg1-*ku70*(3′UTR)-*nptII*-*ku70*(5′UTR) for cloning**	
F *ku70*(5**′**UTR)	GCGGTACTAGTCGCCACCACGGTAGCGGTA
R *ku70*(5**′**UTR)	ACAAGATCTTGACGTCCTTTGGATGTTGCT
F *nptII*	ATGATTGAACAAGATGGATTGC
R *nptII*	TCAGAAGAACTCGTCAAGAAG
F *ku70*(3**′**UTR)	GCGGATCCAGTGTACTAGCGTGACGCTAGA
R *ku70*(3**′**UTR)	CTGGCGGTACCGCTGGCCGACCCACTCGTA
**pAg1-*cnb1*(3′UTR)-*NAT1*-*cnb1*(5′UTR) for cloning**	
F *cnb1*(5**′**UTR)	TGAACTAGTCCGTGATCTGCTGCACGTTCGGGTCC
R *cnb1*(5**′**UTR)	AAAGGGCCCAAGATCTAGTGATAGATGTGTGGAGA
F *cnb1*(3**′**UTR)	CTGGGATCCGCGCGCACACACGGATGTGAGCGTAA
R *cnb1*(3**′**UTR)	CGCGGTACCACTGTTCACCTCTGGCATTGTTACGA
**Genotyping**	
**Primers-1 for *ku70* genotyping**	
F* ku70* gene locus	TCGAGGTCGCGACTTTGTTATTGCCAGGTCCTGA
R *ku70* gene locus	AGAGCTGCGATCGTGGGCTGATCCGTCC
**Primers-2 for *ku70* genotyping**	
F* ku70* gene ORF	TTTCAGCAACTCCGTCAGATCAGCGCCGAAGACA
R* ku70* gene ORF	ATCTGCGAAAGAGCGGCCGGGCC
**Primers-3 for *ku70* genotyping**	
F* ku70* gene outside	TTCATCGACTGTGGCCGGCTGGGTGTGG
R* ku70* gene outside	GGACGAGATGGCCGGGGACCGGCTC
**Primers-1 for *cnb1* genotyping**	
F* cnb1* gene locus	GGAGTGAAGAAGGGCAGAGAGCAACAACAGCGGT
R* cnb1* gene locus	CCGTGATCGCATGGGGCGTGCACAAAGTG
**Primers-2 for *cnb1* genotyping**	
F* cnb1* gene ORF	CGGCTCGGGTACGGTAGACTTCCAGGAGTTTGTCG
R* cnb1* gene ORF	AACAGGTCCTCGAGCGTCATCTGCTTGACGATGT
**Primers-3for*cnb1*genotyping**	
F* cnb1* gene outside	GGACGGCGAGCAGGCGCTCTACATGAGC
R* cnb1* gene outside	CTGAGTCCCATCGGCCCTTGCCTTCAAGCTACC
**Amplification of *cnb1* cassette for electroporation**	
F* cnb1*-cassette	CCGTGATCTGCTGCACGTTCGGGTCCG
R* cnb1*-cassette	CTGTTCACCTCTGGCTACGACCCCCTCCTC

To prepare competent cells for electroporation, *T. asahii* MPU129 strain was spread on a Sabouraud dextrose agar plate and cultured at 27 °C for 3 days. *T. asahii* cells on the agar were suspended by physiologic saline solution (2 ml), and the suspension was transferred to a 1.5-ml tube. The fungal cells were collected by centrifugation at 8000 rpm for 3 min (TOMY-MX100, TOMY Digital Biology Co. Ltd, Tokyo, Japan) and suspended by adding 1 ml of ice-cold water and centrifuged at 8000 rpm for 3 min. This washing process was repeated 4 times. The washed cells were suspended by adding 1 ml of 1.2 M sorbitol solution and centrifuged at 8000 rpm for 3 min. The obtained fungal cells were suspended with 0.2 ml of 1.2 M sorbitol solution as competent cells. The PCR-amplified 5′-UTR (*cnb1*) -NAT1-3′-UTR (*cnb1*) fragment (180 ng/2 µl) was added to the *T. asahii* competent cells (40 µl) and placed on ice for 15 min. The suspension was added to a 0.2-cm gap cuvette (Bio-Rad Laboratories, Inc.) and electroporated (Time constant protocol: 1800 V, 5 ms) using a Gene Pulser Xcell (Bio-Rad Laboratories, Inc.). The cells were suspended by adding 500 µl YPD containing 0.6 M sorbitol and incubated at 27 °C for 3 h. After incubation, the cells were collected by centrifugation at 10,000 rpm for 5 min and suspended in 100 µl of physiologic saline solution and applied to Sabouraud dextrose agar containing nourseothricin (300 µg/ml). The cells were incubated at 27 °C for 3 days and the growing colonies were isolated as *cnb1* gene-deficient strain candidates. Introduction of the mutation into the genome of the candidate strains was confirmed by PCR using the primers shown in Table 2 and the extracted genome as a template DNA.

### Statistical analysis

All experiments were performed at least twice and the representative results were shown. The significance of differences between groups in silkworm infection experiments was calculated by the log-rank test based on the curves by the Kaplan–Meier method using Prism 9.1.2.

## Supplementary Information

Supplementary Information.

## Data Availability

The datasets generated during the current study are available from the corresponding author on reasonable request.
