# Supplementary material for: Development of an efficient gene-targeting system for elucidating infection mechanisms of the fungal pathogen Trichosporon asahii
Source: Sci Rep. 2021 Sep 14;11:18270. doi: 10.1038/s41598-021-97287-3 (PMC8440527; doi:10.1038/s41598-021-97287-3)
Supplement: Supplementary file 1 — Supplementary Information. [file 41598_2021_97287_MOESM1_ESM.docx]

**Supplementary information**

**Development of an efficient gene-targeting system for elucidating infection mechanisms of the fungal pathogen *Trichosporon asahii***

**Yasuhiko Matsumoto^1*^, Tae Nagamachi^1^, Asami Yoshikawa^1^, Hideki Yamazaki^1^, Yusuke Yamasaki^1^, Tsuyoshi Yamada^2,3^, and Takashi Sugita^1^**

^1^Department of Microbiology, Meiji Pharmaceutical University, 2-522-1, Noshio, Kiyose, Tokyo 204-8588, Japan.

^2^Teikyo University Institute of Medical Mycology, 359 Otsuka, Hachioji, Tokyo 192-0395, Japan.

^3^Asia International Institute of Infectious Disease Control, Teikyo University, 2-11-1, Kaga, Itabashi-ku, Tokyo, 173-8605, Japan

*Address correspondence to: Dr. Yasuhiko Matsumoto, Department of Microbiology, Meiji Pharmaceutical University, 2-522-1, Noshio, Kiyose, Tokyo 204-8588, Japan, Tel: +81-42-495-8745, e-mail: ymatsumoto@my-pharm.ac.jp.


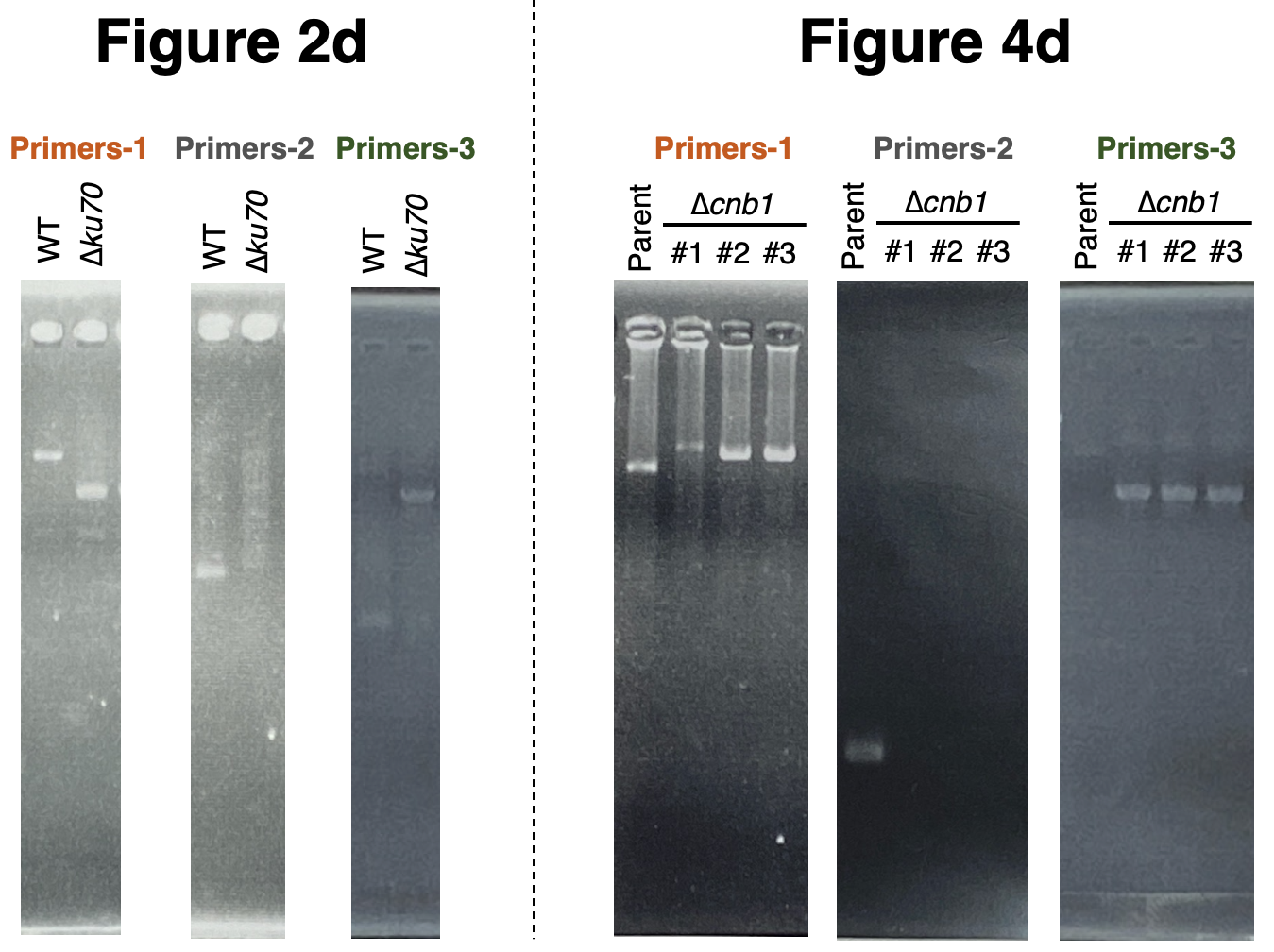


**Supplementary Figure 1 Full-length blots of Figure 2d and Figure 4d.**
